# Mechanisms of Phosphine Toxicity

**DOI:** 10.1155/2011/494168

**Published:** 2011-04-28

**Authors:** Nisa S. Nath, Ishita Bhattacharya, Andrew G. Tuck, David I. Schlipalius, Paul R. Ebert

**Affiliations:** ^1^School of Biological Sciences, University of Queensland, St. Lucia, QLD 4072, Australia; ^2^Agri-Science Queensland, Department of Employment Economic Development and Innovation, EcoSciences Precinct, GPO Box 46, Brisbane, QLD 4001, Australia; ^3^Cooperative Research Centre for National Plant Biosecurity, LPO Box 5012, Bruce, ACT 2617, Australia

## Abstract

Fumigation with phosphine gas is by far the most widely used treatment for the protection of stored grain against insect pests. The development of high-level resistance in insects now threatens its continued use. As there is no suitable chemical to replace phosphine, it is essential to understand the mechanisms of phosphine toxicity to increase the effectiveness of resistance management. Because phosphine is such a simple molecule (PH_3_), the chemistry of phosphorus is central to its toxicity. The elements above and below phosphorus in the periodic table are nitrogen (N) and arsenic (As), which also produce toxic hydrides, namely, NH_3_ and AsH_3_. The three hydrides cause related symptoms and similar changes to cellular and organismal physiology, including disruption of the sympathetic nervous system, suppressed energy metabolism and toxic changes to the redox state of the cell. We propose that these three effects are interdependent contributors to phosphine toxicity.

## 1. Discovery and Applications

Phosphine (PH_3_) was discovered in the late 1700s and has been used as a grain fumigant since the 1930s [1]. It is by far the dominant means of controlling pest insects in stored grain and many other stored commodities. Despite the importance of phosphine to global food security, the mechanisms by which it acts are not understood. This review focuses on the toxicology of phosphine, attempting to integrate the chemistry of phosphine with the biochemical and physiological responses to phosphine exposure. Discussed here are mechanisms of toxicity caused by acute exposure that potentially leads to mortality. Other aspects of phosphine action such as regulation of uptake, disruption of reproduction or genotoxicity are not included. Phosphine resistance is not discussed in detail, but may be referred to as required to explain a mechanism of phosphine toxicity. 

Phosphine is widely used as a fumigant [2] as it is gaseous above −88°C with a density of 1.17 times that of air, which allows it to disperse readily during fumigation. Concentrated phosphine is potentially explosive in air and can autoignite at near ambient temperatures. As such, it is typically applied together with carbon dioxide, which limits its flammability. Phosphine is highly toxic to aerobically respiring organisms, but not to anaerobic or metabolically dormant organisms. Thus, it can be used to kill insect pests in grain, without affecting grain viability. The stable breakdown products of phosphine are harmless phosphorus oxides, which become incorporated into normal cellular metabolism as phosphate. The ease of application, together with its effectiveness, lack of residues, and low cost of the chemical, has resulted in its use on nearly all internationally traded grain destined for human consumption. 

Phosphine is also used in the inorganic and organic synthesis of complex chemicals, a field of research that has expanded dramatically in the past four decades [3]. The reactivity and chemical flexibility of phosphorus has led to the development of a wide range of phosphine derivatives in which the hydrogen atoms of phosphine are substituted with either organic or inorganic replacements. These substitutions alter the nucleophilic or electrophilic nature of the phosphorus, modify the rate of reaction or sterically constrain the reactions that occur. Drugs and pesticides have been developed from chemicals produced in this way.

## 2. Chemical Properties of Phosphine

The phosphine molecule consists of a single phosphorus atom and three hydrogen atoms. As such, the chemistry of the molecule is dominated by the chemistry of the element phosphorus, which is key to the toxicity of the molecule. Because elements are arranged in the periodic table according to the periodicity of their chemical properties, it is instructive to compare the toxic mechanisms of the elements located immediately above phosphorus (nitrogen) or below phosphorus (arsenic) in “group 15" of the table. The fact, that the hydrides of each of the three elements, NH_3_, PH_3_ and AsH_3_ are all toxic gases supports the validity of this approach.

Nitrogen is a major component of nucleotides and amino acids as well as a range of cofactors, secondary metabolites and signalling molecules. Despite its many essential biological roles, nitrogen also can be toxic, requiring that its levels be closely regulated [4]. Excreted forms of nitrogen range from ammonia to urea and its derivatives. In contrast to nitrogen, arsenic is highly toxic to most organisms, though some organisms have evolved special mechanisms to avoid it, that is, sequestration as non-toxic derivatives or in special compartments of cells; generation of insoluble complexes or volatile forms; exclusion from the cell via efflux pumping. Despite its toxicity in most instances, arsenic is also an essential micronutrient, serving as a cofactor in methionine biosynthesis [5]. It is also used as the final electron acceptor in energy metabolism of some anaerobically respiring bacteria [6], similar to the way in which other anaerobic bacteria use phosphine [7].

Each of nitrogen, phosphorus and arsenic, has five valence electrons, with two paired electrons in the s orbital and three unpaired electrons in their p orbitals. The three p orbital electrons can directly contribute to three molecular bonds. The s orbital electrons of phosphorus and arsenic also can participate in bond formation. This is often interpreted as promotion of the paired s orbital electrons to the d orbitals of their outer valence shell, facilitating formation of a double bond, as with oxygen, but this is disputed [8]. The alternative is a single bond with considerable charge imbalance between the P and the O, which may contribute to the reactivity of the first oxy derivative of phosphine, H_3_PO.

As a first row element, the valence electrons of nitrogen are shielded from the positively charged nucleus by only the two 1s electrons. As a result, nitrogen is much more electronegative than the other group 15 elements. In the hydride (NH_3_), ammonia, this results in a partial negative charge on the nitrogen, thereby increasing its affinity for protons in aqueous solution (NH_4_^+^). In contrast, the larger elements, phosphorus and arsenic, are not so electronegative and do not form cationic hydrides to an appreciable degree under physiological conditions.

In biological tissues, phosphorus is present in the fully oxidised form, phosphate, which is strongly thermodynamically favoured over its more reduced counterparts [3], even at a physiological pH [9]. Nitrogen exists in a wide range of oxidised forms in tissues, from ammonia to nitrate, most of which are not particularly strongly oxidising or reducing under physiological conditions. Arsine (AsH_3_) is oxidised to arsenite (AsO_3_^3−^) in the cell, which reacts with reduced sulfhydryls. Arsenate (AsO_4_^3−^) is toxic in its own right as it can act as a phosphate analogue, replacing phosphate in the cell. Arsenate esters are unstable, however, as arsenate is readily reduced to arsenite. Thus, of the three elements, only phosphorus is consistently fully oxidised under physiological conditions.

Phosphine, which is the fully reduced form of phosphorus, oxidises slowly in weak acid. In tissues, this process is accelerated, with the relatively stable intermediate oxyacids, hypophosphite (H_2_PO_2_^−^), and phosphite (HPO_3_^2−^), being produced in addition to phosphate (PO_4_^3−^). None of the stable oxidation products of phosphine are significantly toxic. The singly oxygenated derivative (H_3_PO) is extremely unstable and is not observed either *in vitro* or *in vivo *[10–12]. In H_3_PO, the oxygen carries a partial negative charge and the phosphorus a partial positive charge [8]. The reactivity of the molecule is likely due to the polarity of the molecule, which converts the phosphorus into an electrophile. It is interesting to note that phosphine is not toxic to organisms that are not actively aerobically respiring [13]. In these organisms, phosphine is not appreciably oxidised. Because phosphine itself is not toxic when it is not oxidised and the stable oxidation products are themselves not toxic, understanding the formation and activity of the proposed unstable intermediate, H_3_PO, would seem to be key to understanding the toxicity of phosphine.

## 3. Physiological Alterations and Clinical Symptoms of Phosphine Toxicity

Phosphine toxicology has been explored in a variety of organisms. Because of its use as a grain fumigant for the control of insect pests, it was first studied in these target animals. Recently, these studies have been extended to the model invertebrate organism, *C. elegans*. Invertebrate studies usually involve fumigation at fairly low concentrations for a period of 20 to 48 hours. This approximates the situation when grain is fumigated, which involves exposure to low concentrations of phosphine gas over an extended period of time (i.e., days to weeks). 

In contrast, vertebrates typically experience acute exposure to extremely high concentrations of phosphine. This may be the result of workplace accidents or attempted suicide in humans, whereas in the case of rodents, it is through the ingestion of zinc phosphide treated grain, which is used as a rodenticide. Thus, unlike studies on invertebrates, laboratory studies on either rats or mammalian cell lines typically involve exposure to aluminium or zinc phosphide as a solid formulation or in solution at extremely high concentrations. While this reflects the clinical situation of attempted suicide, as well as the ingestion of rodenticide, it complicates direct comparison of phosphine toxicology between vertebrate and invertebrate. Furthermore, the toxicology reported in vertebrates often includes exposure to the conjugate metals and possibly ammonium and carbonate that may be included in commercial phosphide formulations.

Despite the different exposure conditions and physiology between invertebrate and vertebrate organisms, there are some obvious similarities in the response to phosphine [14, 15]. The common symptoms include an initial state of agitation/hyperactivity followed by lethargy, a decrease in metabolic output and an increase in oxidative stress. Insects and nematodes also become anaesthetised, whereas vertebrates exhibit pulmonary oedema, inhibited oxygen transport, metabolic acidosis, hypotension, and cardiac failure. Individuals who do not die of cardiac failure commonly die of hepatic failure. Similar symptoms are caused by exposure to acutely toxic doses of arsenic and its oxidised derivatives, which result in metabolic suppression and the generation of oxidative stress [16, 17]. Oxidised derivatives of nitrogen have many effects, including hypotension in the case of nitric oxide and pulmonary oedema in the case of other oxides of nitrogen [18].

## 4. Three Potentially Interrelated Aspects of Phosphine Toxicity

The preceding symptoms, as well as the biochemical and physiological changes that occur in response to phosphine exposure, can be grouped into three possible categories of phosphine action neural, metabolic, and redox related. Research relevant to each component of phosphine toxicity will be discussed independently, though they are likely to be causally related. The review will conclude with a pictorial summary of phosphine sites of action that also incorporates known molecular targets of bioactive derivatives of arsenic and nitrogen.

## 5. Neural/Behavioural Aspects of Phosphine Toxicity

Phosphine initially causes agitation followed by convulsions in humans and hyperactivity followed by twitching in nonhuman animals, which may be mediated by the same factors. This is followed by lethargy in humans and what has been referred to as narcosis or anaesthesia in animals. There is evidence that phosphine increases acetylcholine neurotransmission by suppressing acetylcholine esterase [19, 20]. Because acetylcholine is an excitatory neurotransmitter and the role of the esterase is to attenuate acetylcholine signalling, exposure to phosphine would be expected to inhibit the attenuation. The net result would be overactive acetylcholine signalling, which would most likely be expressed as hyperactivity and in extreme cases, excitotoxicity. There is direct evidence that acute phosphine exposure leads to a decrease of insect acetylcholine esterase activity, both *in vitro* and *in vivo*, though the *in vitro* concentration of phosphine that was used was quite high [21]. 

The strongest evidence that excessive acetylcholine signalling via inhibition of the esterase contributes to the toxicity of phosphine is a pharmacological study performed on rats [22]. In this study, pralidoxime, a competitive antagonist of chemical inhibitors of acetylcholinesterase, and atropine, a muscarinic receptor antagonist, were tested for their ability to protect rats that had been exposed to phosphine. Under a phosphine dose that killed the control rats, the combined treatment with pralidoxime and atropine extended the lifespan of nine of the rats 2.5-fold and prevented altogether, the phosphine-induced mortality of the remaining six rats. In this context, it is interesting that arsenite also inhibits acetylcholinesterase [23]. 

Grain fumigation typically relies on exposure to moderate doses of phosphine for extended periods of time, which suggests that the acetylcholine-mediated toxicity of phosphine is not due to acute neural excitotoxicity. This is not surprising as atropine affects G-protein coupled (i.e., muscarinic) acetylcholine signalling, rather than the ligand gated ion channel receptors that are responsible for acetylcholine-mediated excitotoxicity. Muscarinic signalling in vertebrates participates in regulation of the sympathetic nervous system, which, among other things, sets the metabolic rate of the organism. An intriguing study in *C. elegans* suggests muscarinic signalling plays a similar role in invertebrates [24]. In that report, activation of muscarinic signalling induced emergence from the diapause state that is characterised by a low metabolic rate. Atropine, by inhibiting muscarinic signalling, was able to prevent metabolic reactivation from diapause. This suggests that phosphine-mediated activation of acetylcholine signalling results in an increase in metabolic output and perhaps metabolic demand as would be expected on emergence from diapause. The mechanism whereby atropine prevents phosphine-mediated mortality would likely be the reverse of this effect. This conjecture is strongly supported by the clear demonstration that a chronic decrease in metabolic rate results in phosphine resistance [25], whereas an increase in metabolic demand causes hypersensitivity toward phosphine [26].

## 6. Mitochondrial Physiology

There is strong evidence that phosphine disrupts energy metabolism, particularly mitochondrial function. Because of this, relevant aspects of mitochondrial function will be described here, prior to discussion of specific interactions between mitochondrial energy metabolism and phosphine. 

The mitochondrial membrane structure is critical to the energy metabolism of the cell. The mitochondrion is a double membrane bound organelle with a permeable outer membrane but a highly impermeable mitochondrial inner membrane (MIM). This ensures the controlled passage of molecules across the MIM between the intermembrane space and the central mitochondrial matrix, but a more relaxed exchange between the intermembrane space and the cytoplasm. The transfer of molecules across the MIM as well as key metabolic reactions, including the phosphorylation of ADP to ATP, are dependent on the potential energy stored in a proton gradient across the inner membrane. 

**Figure 1:**
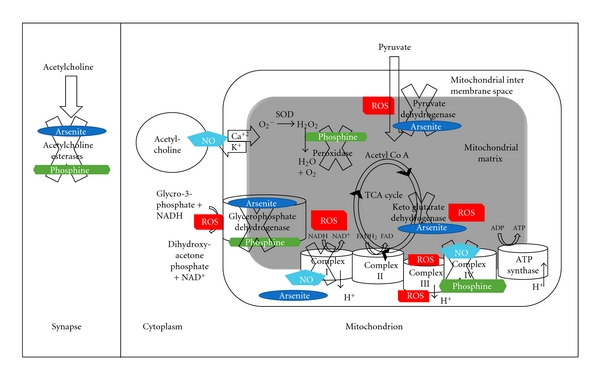
Interaction of phosphine with enzymes involved in metabolic processes and acetylcholine signalling. Sites of interaction with arsenite and nitric oxide (NO) are also shown: phosphine (green), arsenite (dark blue), and nitric oxide (light blue). Sites of ROS generation (red) are indicated as well. The cross behind the names of targeted enzymes indicates that they are inhibited. The potassium and calcium currents are regulated by acetylcholine via NO. Ca^2+^ triggers the release of acetylcholine from vesicles in the cytoplasm into the neuronal synapse. The acetylcholinesterase degrades acetylcholine, which reduces the strength of neurotransmission. The net effect is that arsenite and phosphine increase acetylcholine signalling by inhibiting the esterase. FAD/FADH2 (Flavin adenine dinucleotide oxidised/reduced), NAD^+^/NADH (nicotinamide adenine dinucleotide oxidised/reduced), ADP/ATP (adenosine di/tri nucleotide), NO (nitric oxide), ROS (reactive oxygen species), and TCA (tricarboxylic acid).

The mitochondrial membrane potential is maintained by the electron transport chain (ETC), which is embedded within the MIM (Figure 1). The ETC receives high energy electrons, predominantly from the tricarboxylic acid (TCA) cycle in the matrix, glycolysis in the cytoplasm, and beta oxidation of fats. The energy of these electrons is extracted sequentially by three protein complexes, I, III, and IV, which transport protons from the matrix to the intermembrane space, thereby creating a difference in electrochemical potential between the mitochondrial matrix and the mitochondrial intermembrane space. Complex I receives electrons from NADH, whereas complex II, an alternative electron acceptor that does not pump protons, receives electrons from succinate. These electrons travel via the small molecule electron carrier, coenzyme Q, on to complex III. The electrons are then carried from complex III to Complex IV via cytochrome C. At complex IV, the cell is protected from the potentially dangerous residual energy of the electrons by transfer to the final electron acceptor, molecular oxygen, to generate H_2_O. 

NADH is also produced during glycolysis and other metabolic processes in the cytosol. The NADH in the cytoplasm is unable to enter the matrix and hence cannot be used by Complex I directly. Instead, the electrons from cytoplasmic NADH are passed to coenzyme Q within the MIM by the enzyme glycerophosphate dehydrogenase. Glycerophosphate dehydrogenase may have a very significant role to play in phosphine toxicity as will be discussed later.

## 7. Contribution of Metabolic Rate to Phosphine Toxicity

Phosphine inhibits respiration in rat liver mitochondria [27], insect mitochondria [28–31], and intact nematodes [25]. The degree of suppression is most significant when mitochondrial respiration is activated by the addition of either chemical uncoupler or precursors to ATP synthesis. In contrast, little or no inhibition of resting state respiration was observed unless the mitochondria were broken open by sonication [28]. This suggests that the inner membrane provides a barrier to phosphine uptake that can be circumvented either by physical disruption of the membrane or by activation of transport across the MIM. 

A careful analysis using specific substrates revealed Complex IV, cytochrome c oxidase, to be the primary site of *in vitro* interaction between phosphine and the electron transfer chain [27, 28]. This work was extended by Kashi and Chefurka [32] who looked at the oxidation state of the two cytochromes within complex IV. They found that phosphine treatment caused cytochrome a, but not cytochrome a_3_ to become highly reduced [32]. While this demonstrated a differential interaction between phosphine and the two cytochromes, it did not reveal the nature of the interaction or whether the state of reduction of the cytochromes was the direct cause of phosphine toxicity. 

The direct disruption of mitochondrial respiration by phosphine initially seemed to suggest that energy insufficiency was the cause of mortality due to phosphine exposure, but seemingly contradictory results were obtained depending on whether mitochondria were isolated prior to phosphine exposure or were isolated from insects previously exposed to phosphine. Inhibition of oxidative respiration by phosphine occurred when isolated mitochondria were treated, but the effect was reduced or absent when live insects were treated prior to mitochondrial isolation [31, 33]. It is important to note that the exposure of the live insects to phosphine was sufficient to cause obvious narcosis prior to mitochondrial isolation, so the phosphine had had time to penetrate the tissues and cause a physiological response [29, 33]. It is also important to note that the *in vitro* suppression of mitochondrial function was stable. As such, the inability to observe a similar effect when animals were treated *in vivo* was not due to the time required to isolate the mitochondria following phosphine exposure [33]. It seems that *in vitro* inhibition occurs by direct modification of the ETC that does not occur when living animals are exposed to phosphine.

Furthermore, respiration in mitochondria isolated from a genetically resistant strain of insect was just as strongly inhibited by *in vitro *exposure to phosphine as was respiration in mitochondria isolated from a sensitive strain [33]. Thus, *in vitro* modification to ETC function did not seem to correlate with the resistance mechanism of that particular resistant strain of insects [33]. A separate study found that oxygen consumption in live insects decreased after a period of four-hours exposure to phosphine, but only in resistant insects [34]. Thus, respiration rates in live insects are related to the resistance phenotype, but a four hour period of physiological adaptation is required. In contrast, disruption of mitochondrial function by phosphine was unrelated to resistance when the tests were carried out *in vitro*. It is as if the physiological state of the living animal is an essential component of the resistance mechanism. 

While the *in vitro* experiments demonstrate that phosphine can interact with complex IV, there is no indication that *in vitro* results can be used to explain either phosphine toxicity or resistance. There is, however, a compelling case for a relationship between energy metabolism and phosphine toxicity in living animals [35], though one must consider both energy demand and energy generation capacity when interpreting the results. For example, insect pests of stored grain are unaffected by phosphine when they are exposed under hypoxic conditions [36, 37], presumably due to suppressed metabolic demand coupled to the suppressed aerobic respiration. Similarly, tolerance of insects to phosphine seems to be associated with the degree of oxygen uptake [38]. In nematodes, genetic disruptions to the mitochondrial ETC, the primary site of oxidative respiration, are associated with resistance toward phosphine [25]. Resistance in a mutant strain of *C. elegans*, *pre-33*, is associated with a respiration rate that is suppressed to about 25% of normal [25]. The same strain does not exhibit enhanced sensitivity toward phosphine under conditions of hyperoxia [39]. The slow growth and decrease in fecundity caused by ETC disruption or the *pre-33* mutation indicate that metabolic demand is suppressed to compensate for the slow metabolism. Conditions that activate metabolism and probably increase metabolic demand as well, increase sensitivity toward phosphine. Thus, nematodes under hyperoxic conditions [39] and insects at increased temperature [40] are more sensitive to phosphine. Mitochondrial uncouplers, which directly increase metabolic demand, greatly enhance phosphine toxicity [26]. 

Because most of the preceding examples do not distinguish between the effect of metabolic rate and energy demand, two interpretations are possible. Either the increased rate of metabolism itself is directly responsible for phosphine toxicity or energy insufficiency causes a metabolic crisis leading to death. The example of treatment with mitochondrial uncouplers [26] does indicate that artificially increasing energy demand can increase sensitivity toward phosphine. Even if this is not the mechanism of toxicity under normal conditions, it does provide a possible resistance management strategy. 

Two examples of a phosphine-induced metabolic crisis have been reported—one in rat [41] and the other in *C. elegans* [25]. Rats treated with phosphine synthesised glucose in the liver but increased rates of glycolysis in the brain tissue. While the authors did not measure oxygen consumption, they suggested that a decrease in aerobic respiration forced the energy needs to be met by the much less efficient anaerobic respiration. A sharp drop in plasma glucose levels supported the interpretation of an ensuing metabolic crisis [41]. This response may be similar to that observed in a sensitive strain of nematode in which exposure to phosphine caused a sharp drop in oxygen consumption [25]. 

It is difficult to compare results directly, without knowledge of the resistance genes themselves, but a unique result was obtained with *C. elegans*. In this organism, the phosphine resistant mutant *pre-33* has a constitutively suppressed rate of oxidative respiration [25] and does not exhibit enhanced sensitivity toward phosphine under conditions of hyperoxia [39]. Nonmutant animals initially have a high rate of respiration, but this crashes within one hour to the constitutively suppressed rate of the resistant mutant [25]. Comparison of the different responses between insect and nematode suggest that in insects, a controlled suppression of metabolism is associated with resistance, whereas in the nematode, preadaptation of the resistant strain at a low metabolic rate prevents a phosphine-induced metabolic crash. Clarification awaits identification of resistance genes and molecular comparisons between species as it is clear that at least two distinct resistance mechanism exist in each of insects [42, 43] and nematodes [39].

## 8. Contribution of Oxidative Stress to Phosphine Toxicity

Cellular oxidative stress is caused by the generation of reactive oxygen species (ROS), predominantly superoxide (O_2_^·−^), hydrogen peroxide (H_2_O_2_), and reactive nitrogen species (RNS), predominantly nitric oxide (^·^NO) and peroxynitrite (OONO^−^), as byproducts of a subset of enzymes that engage in electron transfer. These ROS/RNS are potentially highly damaging to biological macromolecules, ultimately leading to cell death. Under normal conditions, the primary source of ROS/RNS is the mitochondrial ETC [44]. Even if electrons flow freely through the ETC, a small amount of superoxide is generated through the inappropriate transfer of electrons to molecular oxygen at complexes I and III. If the flow of electrons is impeded, however, the inadvertent production of superoxide can become very significant [45]. As phosphine is known to inhibit cytochrome c oxidase, phosphine-induced generation of ROS provides a strong alternative model to mortality caused by energy insufficiency. The decrease in ROS generation associated with decreased energy metabolism may explain how metabolic suppression can enhance tolerance toward phosphine.

In fact, glycerophosphate dehydrogenase [46] is inhibited by phosphine, resulting in stimulation of hydrogen generation [46]. These authors did not inhibit the enzyme superoxide dismutase to prevent the conversion of superoxide to hydrogen peroxide, so at least some of the ROS that they measured as hydrogen peroxide could have initially been produced as superoxide. This is in fact observed in *Drosophila melanogaster*, in which inhibition of the ETC results in significant superoxide production from glycerophosphate dehydrogenase [47]. Regardless of the specific ROS produced or the site of its production, this example provides a clear and immediate link between metabolic inhibition by phosphine and the generation of oxidative stress.

It has been noted that fumigation moderately induces superoxide dismutase, but inhibits the activity of peroxidase and catalase [29, 46, 48]. The net effect is the enhanced conversion of superoxide to hydrogen peroxide by the enzyme superoxide dismutase, but lack of detoxification of the resulting hydrogen peroxide by conversion to water via catalase or peroxidase. Phosphine has also been shown to react chemically with hydrogen peroxide to generate an even more reactive oxygen species, the hydroxyl radical [49]. 

Phosphine-induced oxidative damage to macromolecules has been demonstrated in insects [50], nematodes [26], mammalian cell lines [51], and in rats [52]. A detailed analysis of the types of ROS induced by phosphine and the roles of specific protectants identified hydrogen peroxide as the most significant ROS and glutathione as the strongest protective antioxidant [51]. Glutathione not only protected against oxidative damage, but enhanced cell survival as well. The authors stated that endogenous glutathione levels did not change over the treatment period and that glutathione was likely acting as an antioxidant as it did not inactivate the phosphine directly [51]. While an extremely high concentration of aluminium phosphide was used in this study, the results are supported by observations that treating rats [53] or nematodes [26] with glutathione depleting chemicals results in increased sensitivity toward phosphine. 

Even though there is significant evidence in support of a role for oxidative stress in phosphine toxicity, there is some contradictory evidence and some critical experiments that remain to be carried out. The most significant gap in the literature is a clear demonstration that oxidative stress is the cause of phosphine-induced mortality in animals. It is clear that antioxidants, most notably melatonin, can prevent most of the oxidative damage induced in a range of tissues in rat [52, 54] and that melatonin can protectively maintain the levels of glutathione [55]. What has not been reported is the relationship between the level of macromolecular oxidative damage and phosphine-induced mortality. Such experiments are straightforward and should be carried out as a priority.

Phosphine is a reducing agent that can complex with metal ion cofactors at the active site of enzymes, which is the basis of phosphine-mediated inhibition of enzymes such as cytochrome c oxidase and catalase [56]. As a reducing agent, phosphine may also reduce disulfides. The capacity of phosphine to reduce a disulfide was reported in 1970, but the reaction was found to be extremely slow in a neutral solution [11]. At the time, disulfide bonds were believed to simply be structural, so no attempt was made to test catalytic disulfides. More recently, direct redox interaction between phosphine and cysteine at the reactive disulfide of glutathione reductase was proposed to be the mechanism by which phosphine inhibited the enzyme [57]. The possibility that phosphine acts at catalytic cysteine residues in proteins remains to be explored.

It is interesting to note that both nitrogen and arsenic carry out redox reactions with cysteine amino acids in proteins. For instance, when nitric oxide is activated by superoxide to form peroxynitrite, it is capable of nitrosylating reduced cysteine residues [58]. In the case of arsenic, the active form of the element in the cell is arsenite, which also can link covalently to target sulfhydryls [17]. In contrast to the other two elements, phosphine is strongly thermodynamically favoured to act as a reducing agent [9, 59]. In this regard, phosphine itself (or a proposed but highly unstable hydroxy phosphine derivative) [10] is the toxic form of the element, whereas the more oxidised oxyacids, hypophosphite, phosphite, and phosphate are not toxic. Thus, while phosphine may act on cysteine residues *in vivo*, the mechanism of the interaction may differ significantly from that of nitrogen and arsenic. As with nitric oxide, however, activation of phosphine by locally generated ROS is a possibility.

## 9. Comparison of the Biological Activities of the Elements N, P, and As

Figure 1 shows the known biochemical targets of phosphine. It is intriguing that the three elements all form toxic, gaseous hydrides [60, 61], but that the redox properties of their oxygenated derivatives at physiological conditions differ considerably. The nitrogen derivatives are free radicals, oxidants, or reductants, whereas phosphine and its oxyacid derivatives other than phosphate are strongly reducing compounds. Arsenic is most abundant in the cell as arsenite, but can mimic phosphate when fully oxidised to arsenate [62]. The sites of action of the oxides of nitrogen and arsenic have been included in the figure at the specific sites and biochemical processes they are known to disrupt. From the figure, it is apparent that while many of the same processes are influenced by the three elements, the details differ. The influence of phosphine as well as the oxides of nitrogen and arsenic on acetylcholine signalling is also shown in Figure 1.

## 10. Summary

The muscarinic acetylcholine antagonist atropine protects rats against phosphine exposure [22], indicating that acetylcholine signalling is an important component of phosphine toxicity. As a regulator of the parasympathetic nervous system in mammals and an activator of the metabolic pathways required for emergence from diapause in *C. elegans* [24], muscarinic signalling is well-placed to be a significant neuroendocrine regulator of metabolism. Each of the three elements, N, P, and As, influences acetylcholine signalling but in unique ways. Nitrogen, as nitric oxide, is a direct mediator of muscarinic signalling [63]. Arsenite and phosphine both inhibit acetylcholine esterase [20–23]. The arsenical metabolic derivative, dimethyl arsenate, forms a covalent adduct with a serine side chain [64], whereas the mechanism of phosphine inactivation of acetylcholine esterase is unknown. 

Altered metabolism is key to phosphine toxicity, but this may either be a direct effect as through induction of a metabolic crisis or an indirect effect through an increase in ROS generation or some other metabolic process. Nitric oxide and phosphine both act at complex IV of the electron transport chain to inhibit electron flow through the ETC, though the phosphine effect is more significant *in vitro* than *in vivo*. Arsenite and nitric oxide also act at complex I [65–67]. Other metabolic enzymes are also targeted by the three related elements and in most cases ROS are generated as a result. 

In some cases, suppression of oxidative metabolism is associated with resistance toward phosphine as occurs under hypoxic conditions. This is likely associated with a suppression of energy demand, which prevents a metabolic crisis, though an equally valid possibility is that fewer ROS are generated. In other cases, a sharp drop in respiration following exposure to phosphine, as in sensitive rats and nematodes has been attributed to a metabolic crisis. Stimulation of energy demand through the chemical uncoupling of ATP synthesis from the flow of electrons through the ETC enhances the toxicity of phosphine [26]. This likely results from an induced metabolic crisis, though the possibility of increased ROS generation cannot be excluded. 

Three very different, but potentially interrelated mechanisms of action have been discussed in this review. The first of these is signalling through acetylcholine, which is not only an excitatory neurotransmitter, but also the primary regulator of metabolism through the parasympathetic nervous system. Acetylcholine toxicity is likely mediated through its role as a metabolic regulator. Ultimately, toxicity may be the result of a metabolic crisis, the generation of reactive oxygen species or some other redox activity of phosphine.
